# Circulating neutrophil extracellular traps in cats with hypertrophic cardiomyopathy and cardiogenic arterial thromboembolism

**DOI:** 10.1111/jvim.16676

**Published:** 2023-03-23

**Authors:** Ronald H. L. Li, Arianne Fabella, Nghi Nguyen, Joanna L. Kaplan, Eric Ontiveros, Joshua A. Stern

**Affiliations:** ^1^ Department of Surgical and Radiological Sciences, School of Veterinary Medicine University of California, Davis Davis California USA; ^2^ Veterinary Medical Teaching Hospital, School of Veterinary Medicine University of California, Davis Davis California USA; ^3^ Department of Medicine and Epidemiology, School of Veterinary Medicine University of California, Davis Davis California USA

**Keywords:** cell‐free DNA, fragment size, NETosis, peptidyl arginine deiminase 4

## Abstract

**Background:**

Cats with hypertrophic cardiomyopathy (HCM) are at risk of cardiogenic arterial thromboembolism (CATE). Neutrophil extracellular traps (NETs) may be a potential biomarker and therapeutic target for cardiomyopathy in cats.

**Hypothesis/Objectives:**

Characterize NETs in cats with HCM or CATE. We hypothesized that circulating NETs assessed in the form of cell‐free DNA (cfDNA) and citrullinated histone H3 (citH3) are increased in cats with HCM and CATE and associated with reported predisposing factors for thrombus formation.

**Animals:**

Eighty‐five cats including client‐owned cats with HCM and CATE and staff‐ and student‐owned clinically healthy cats without HCM.

**Methods:**

After echocardiographic evaluations, NETs were measured as cfDNA and citH3.

**Results:**

Cats with CATE had significant increases in cfDNA (11.2 ng/μL; interquartile range [IQR], 8.1 to 29.6) compared to those without HCM (8.2 ng/μL; IQR, 5.7 to 11.7 μL; *P* = .01) and were responsible for 75% to 83% of cases with cfDNA fragments sized 100 to 2000 base pairs. Citrullinated histone 3, detected in 52% of cats with HCM (31.1 ng/mL; IQR, 16.9 to 29.8), was significantly lower than in those with CATE (48.2 ng/mL; IQR, 34.2 to 60.2; *P* = .007). The citH3 concentrations correlated significantly with reported risk factors of CATE, such as left atrial auricular velocity.

**Conclusions and Clinical Importance:**

Neutrophil extracellualr traps, especially citH3, are increased in cats with HCM and CATE. They may serve as a novel therapeutic target and biomarker of thrombosis in cats with HCM.

**Abbreviations**
bp: base pairs
CATE: cardiogenic arterial thromboembolism
cfDNA: cell‐free DNA
CHF: congestive heart failure
citH3: citrullinated histone H3
HCM: hypertrophic cardiomyopathy
LA: left atrium
LA:Ao: left atrium to aortic root ratio
LAA: left atrial appendage
LAu V: left atrial auricular velocity
NETs: neutrophil extracellular traps
PAD4: peptidyl arginine deiminase 4
SEC: spontaneous echocardiographic contrast
vWF: von Willebrand factor

## 1 INTRODUCTION

Hypertrophic cardiomyopathy (HCM) is the most common cardiac disease in cats affecting approximately 15% of the cat population, and characterized by highly variable disease outcomes from subclinical to severe morbidity and mortality. Cats with HCM that develop clinical signs usually succumb to congestive heart failure (CHF), fatal arrhythmias, or cardiogenic arterial thromboembolism (CATE). Recent studies identified CATE as a major contributor to morbidity and mortality in cats with HCM with an incidence of 11.3% in 1008 cats with HCM.^1^ This finding suggests that the prevalence of CATE was previously underreported.^2^, ^3^ Because cats with CATE often present acutely with extreme pain and no prior warning, CATE remains a distressing emergency for both cat owners and veterinarians, with a mortality rate of up to 67%.^3^, ^4^, ^5^, ^6^ Despite the devastating outcome, clinicians have limited tools to recognize cats at risk of CATE. Echocardiographic assessment of risk for CATE is most commonly employed including the presence of spontaneous echo‐contrast (SEC), left atrial (LA) enlargement and left atrial appendage (LAA) dysfunction.^7^ Identification of these risk factors is complicated by the fact that many cats at risk of CATE appear healthy and do not have auscultatory abnormalities, and hence, may be unlikely to undergo echocardiographic screening.^5^, ^8^ Reliable and accessible techniques to recognize cats with HCM at risk of CATE have yet to be identified.

The pathophysiology of CATE is poorly understood and likely results from dysregulation of each component of Virchow's triad, which includes endothelial dysfunction, hypercoagulability and blood stasis. Formation of neutrophil extracellular traps (NETs), which are web‐like fragments of cell‐free DNA (cfDNA) decorated with histones and neutrophil granular proteins, is an important component of innate immunity.^9^ The prothrombotic properties of NETs, which facilitate microvascular thrombosis, are an important first‐line of defense because they prevent systemic dissemination of pathogens. However, excess circulating cfDNA and NETs proteins can have proinflammatory and prothrombotic consequences. In dogs, neutrophil‐derived cfDNA decreases clot lysis, whereas histones on NETs accelerate clot formation.^10^ Circulating cfDNA fragments also facilitate clot formation by carrying tissue factor and binding to factors XI, XII and high molecular weight kininogen, all critical for promoting thrombin generation.^11^, ^12^, ^13^, ^14^, ^15^ The web‐like scaffold of NETs also fortifies clots by binding to circulating erythrocytes, platelets and fibrin.^16^ Increased cfDNA concentrations and fragment sizes are known to affect fibrin formation and have diagnostic and prognostic value in humans with metastatic neoplasia and ischemic stroke.^17^, ^18^, ^19^ Recently, we identified the presence of NETs as structural components in arterial thrombi from cats with CATE.^20^ Not only were NETs found in all layers of arterial thrombi, but their distribution also varied greatly in relation to their proximity to the initial site of vascular occlusion.^21^ This finding suggests that NETs also may play an important role in the pathogenesis of intracardiac thrombosis and thrombus growth in cats with HCM and CATE. In humans, NETs within coronary thrombi are associated with poor outcome in myocardial infarction and contribute to resistance to thrombolytic therapy.^22^, ^23^ Despite these findings, no studies have yet characterized circulating NETs in cats with HCM or CATE. A better understanding of the role of NETs in thrombosis in cats with HCM is needed. Direct measurement of NETs quantity in blood is not possible and as such independent assessment of NETs components is required. Demonstrating the presence of NETs markers such as cfDNA and citrullinated histones in HCM cats before development of thrombosis may offer diagnostic value in predicting clot formation and could help guide veterinarians toward implementing life‐saving antithrombotic treatments.

We hypothesized that circulating NETs assessed as cfDNA and citrullinated histone H3 (citH3) would be increased in cats with HCM and CATE. We further hypothesized that circulating NETs markers would be increased in cats with HCM before development of thrombosis. In addition, the presence of NETs is expected to be associated with known predisposing factors for CATE in cats. To test our hypotheses, we measured, compared, and characterized concentrations and fragment sizes of circulating cfDNA and citH3 in cats with CATE, in cats with overt HCM (without CATE) and in clinically healthy cats without HCM. We also determined if concentrations of circulating cfDNA and citH3 were associated with probable predisposing factors for thrombus formation in cats with HCM and CATE.

## 2 MATERIALS AND METHODS

### 2.1 Animals and study groups

The study protocol was approved by the Institutional Animal Care and Use Committee at the University of California, Davis (21303) with written informed consent provided for each enrolled patient. Cats in the HCM and CATE groups consisted of client‐owned cats presented to the Veterinary Medical Teaching Hospital, University of California Davis. Cats in the non‐HCM group consisted of staff‐ and student‐owned clinically healthy cats without HCM. All cats underwent complete physical examination, echocardiographic examination, blood pressure measurement by Doppler sphygmomanometer, serum biochemical profile (VC2, Abaxis, Zoetis), CBC (HM5, Abaxis, Zoetis, Parsippany, New Jersey) and measurement of total thyroxine (T4) concentration (VC2, Abaxis, Zoetis, Parsippany, New Jersey). Diagnosis of CATE was made based on physical examination findings and additional diagnostic findings according to the algorithm presented in Table 1.^4^, ^5^, ^18^ Clinical diagnosis of CHF was made based on physical examination findings combined with radiographic evidence of pulmonary edema or pleural effusion on thoracic radiographs and echocardiographic findings of LA or biatrial enlargement.

**TABLE 1:** Diagnostic algorithm for cardiogenic arterial thromboembolism.

• At least one of the following echocardiographic findings is required:
Left atrial enlargement (LA:Ao ≥ 1.6)
Spontaneous echo‐contrast
Intracardiac thrombus
Diastolic LV wall thickness (≥6 mm)
• At least four of the following physical examination findings is required:
Sudden onset of vocalizing
Paralysis or paresis of one or more limbs
Lower motor neuron signs in one or more limbs (absent motor function with absent skin sensation)
Absent femoral and/or dorsal pedal pulses
Pale or cyanotic foot pads/nailbeds of one or more limbs
Firmness of the cranial tibial or gastrocnemius muscles
Low rectal temperature (<37.6°C or <99.7°F)
• Diagnostic findings considered suggestive but not required for diagnosis:
Confirmed aortic or arterial thrombus by abdominal ultrasound
Absence of audible Doppler signal over the artery in question

Cats in the HCM and non‐HCM groups were excluded if they had any of the following: (a) apparent unrelated systemic diseases based on abnormal physical examination findings that otherwise could not be explained by cardiac disease, CHF, or CATE, (b) abnormalities of any serum biochemistry variable or CBC showing evidence of anemia (hematocrit ≤24%), leukocytosis or leukopenia (≤5.5 × 10^9^/L or ≥19.5 × 10^9^/L), or thrombocytopenia (platelet count ≤ 150 × 10^9^/L) confirmed by peripheral blood smear evaluation, (c) systemic hypertension with systolic blood pressure ≥ 160 mmHg, (d) hyperthyroidism (total T4 ≥ 4.8 μg/dL), (e) uncooperative temperament for echocardiography and blood collection, and (f) treatments with an antithrombotic or antiplatelet drug in the past 30 days. Cats in the CATE group were excluded if echocardiographic evidence of cardiomyopathy was absent, and if they were deemed too unstable for echocardiography or blood collection. At home administration of gabapentin (50 to 100 mg PO) before enrollment was permitted. Butorphanol (0.1 to 0.2 mg/kg IV or IM) also was permitted to facilitate safe handling for cats in respiratory distress.

### 2.2 Echocardiography

Transthoracic echocardiographic examinations were performed by a board‐certified veterinary cardiologist or cardiology resident in training under direct supervision of a board‐certified veterinary cardiologist (J.A.S). Cats were gently restrained in right and left lateral recumbency to obtain standard imaging planes as previously described.^24^ For the purpose of the study, the following findings were considered probable risk factors for thrombus formation from previous published disease associations or increased hazard ratios. Evaluated risk factors of CATE included LA enlargement, decreased LAA peak flow velocity, presence of spontaneous echocardiographic contrast (SEC) or intracardiac thrombus or both.^7^, ^25^ Left atrial appendage function was measured by transthoracic pulsed Doppler‐derived maximum left atrial auricular (LAu) velocity as previously described.^7^ Left atrial size was evaluated as a ratio to the aortic root and measured as previously described.^26^ Left atrial enlargement was defined as an absolute 2‐dimensional (2D) long axis value ≥1.6 cm or 2D LA:Ao in short axis ≥ 1.6. All other echocardiographic assessments were performed as previously described.^26^ Diagnosis of HCM required identification of idiopathic regional or global left ventricular wall thickness ≥ 6 mm, as determined by 2D or m‐mode echocardiography avoiding inclusion of moderator band insertion sites and in the absence of systemic disease, systemic hypertension and hyperthyroidism. The presence or absence of any observed intracardiac thrombi along with their location also was recorded. Spontaneous echocardiographic contrast at standard gain setting for the remainder of the examination was recorded as present or absent. Spontaneous echocardiographic contrast was defined as a dynamic and organized swirling pattern visualized within any of the cardiac chambers. All echocardiographic measurements were analyzed by a single investigator (J.A.S) blinded to the study group using off‐line image analysis software (Syngo Dynamics, Siemens).

### 2.3 Blood collection

Blood was drawn from the medial saphenous vein or jugular vein using a 23G butterfly needle within 6 hours of presentation. Blood was drawn from the cephalic or jugular vein in cats with CATE. Whole blood was immediately aliquoted to tubes containing 3.2% trisodium citrate (BD Vacutainer, Franklin Lakes, New Jersey) and lithium heparin (BD Microtainer, Franklin Lakes, New Jersey), placed on ice, and processed within 1 hour of collection. After gentle inversion and inspection for blood clots, a CBC and blood smear evaluation were performed on heparinized whole blood and the remainder of the sample was used for biochemical analysis and total T4 measurement (VS2, Zoetis, Parsippany, New Jersey). A portion of the remnant sample was used for analyzing NETs markers. Citrated and heparinized whole blood then underwent centrifugation (2000 × G, 10 min, 4°C). After extraction of plasma, protease inhibitor cocktail (1× HALT, Thermo Scientific, Waltham, Massachusetts) was added to heparinized plasma to prevent histone degradation, flash‐frozen in liquid nitrogen, and stored at −80°C before analysis.

### 2.4 Isolation and purification of plasma cell free DNA

Citrated plasma first was thawed at room temperature before cfDNA purification using a magnetic bead‐based cleanup kit (QIAamp Minelute ccfDNA mini kit, Qiagen, Germantown, Maryland) as previously described.^27^ The component mixture consisting of proteinase K, magnetic bead suspension and bead binding buffer was adjusted according to the available volume of plasma from each cat. The maximum and minimum volumes of plasma processed for cfDNA purification were 1000 and 500 μL, respectively. To optimize isolated cfDNA concentration, 20 μL of 1× tris‐EDTA buffer was applied directly on the center of column, which underwent 3 additional rounds of elution by reapplying eluate to the column. Eluted cfDNA was stored at 4°C for further analysis.

### 2.5 Quantification of plasma cell free DNA

Concentrations of cfDNA were quantified by spectrophotometry (Nanodrop 2000c, Thermo Fisher Scientific, Waltham, Massachusetts) as previously described.^28^ Tris‐EDTA buffer was used as a blank before sample analysis. Double‐stranded nucleic acid concentration was measured based on absorbance at 280 nm. Purity of cfDNA was determined by a 260/280 ratio of approximately 1.8. Each sample was measured in duplicate and was reanalyzed if the percent difference between the 2 measurements was >5%. Measured cfDNA concentrations then were corrected to plasma volume using the follow calculation:

$$\text{Corrected cfDNA}\;\left(\frac{\mathrm{ng}}{\mathrm{\mu L}}\right)=\frac{1000}{\text{Plasma volume}\;\left(\mathrm{\mu L}\right)}\times \left[\text{cfDNA}\right]\text{measured}.$$

### 2.6 Chip‐based capillary electrophoresis of cell‐free DNA

**FIGURE 1:**
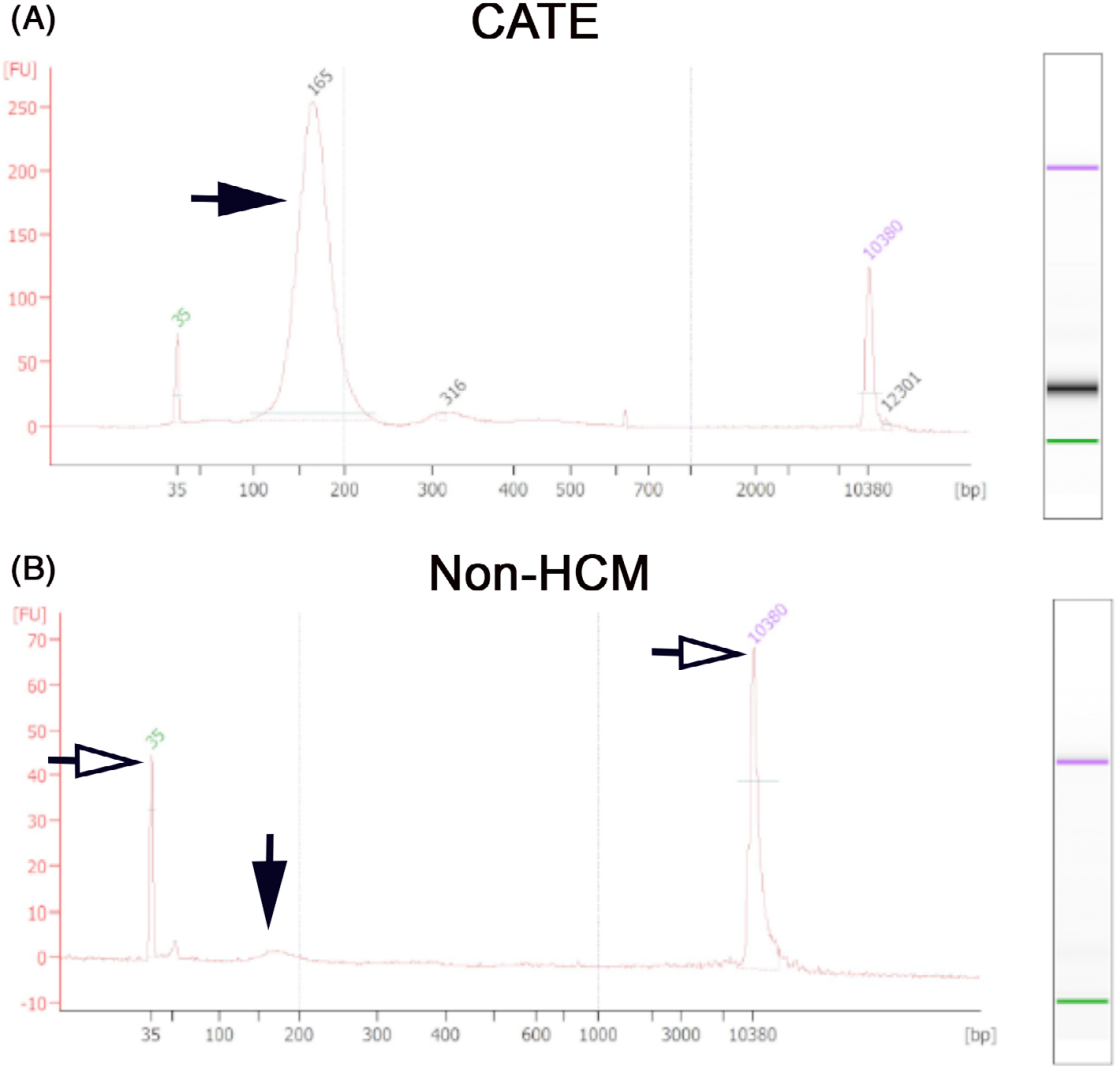
Representative electrograms of plasma cell‐free DNA fragment size and concentrations in plasma from a cat with cardiogenic arterial thromboembolism (CATE) (A) and a cat without hypertrophic cardiomyopathy (non‐HCM) (B). The X‐axis and Y‐axis represent DNA fragment sizes (base pairs; bp) and concentration, based on relative fluorescence units (FU), respectively. (A) A cat with CATE had a high concentration of short fragment size peaking at 165 bp (black solid arrow). (B) Electrogram in a cat without HCM showing a minimal detectable level of cfDNA fragments (black arrow) (lower and upper marker ‐ open/white arrow).

Purified cfDNA was standardized to a final concentration of 5 ng/μL before chip‐based capillary electrophoresis and high sensitivity DNA assay (Agilent 2100 Expert Bioanalyzer, Santa Clara, California). Samples were loaded onto an 11‐well nanochip (Agilent, Santa Clara, California) and analyzed according to the manufacturer's instructions. For samples with cfDNA concentrations ≤5 ng/μL, samples were loaded as is. Briefly, nanochips were primed with 9 μL of gel‐dye mixture followed by loading of standard ladder markers into each well. One microliter of the standard ladder then was loaded into the appropriate labeled well for control, followed by 1 μL of purified samples to each of the remaining wells. The loaded nanochips were placed into the receptacle and the pre‐programed assay was selected to run for 45 min. The standard ladder appeared as 15 distinct peaks on the electropherogram after the first 50 s of the assay. The cfDNA concentrations for DNA fragment length sizes between 35 and 10 380 bp were calculated using commercial software (Agilent 2100 Bioanalyzer software) based on area under the peaks of the electropherogram (Figure 1B). The operator was blinded to treatment groups. Patients with samples that did not have any distinct peaks on electropherogram were reported as undetectable. Fragments of DNA >2000 bp were considered genomic DNA contamination and were not analyzed further.^29^

### 2.7 Semi‐quantitative analysis of plasma citrullinated histone H3 by sodium dodecyl sulfate‐polyacrylamide gel electrophoresis (SDS‐PAGE) and Western blot analysis

To optimize detection of free histones, chromatin was fragmented by first sonicating heparinized plasma using a digital ultrasonic bath (Fisher Scientific, Waltham, Massachusetts) for 30 min, followed by treatment with 80 U/mL of DNase I (New England Biolabs Inc, Ipswich, Massachusetts; overnight incubation at 37°C). Recombinant proteins were subjected to the same conditions to ensure that sonication and DNA digestion did not degrade histone proteins. Plasma protein concentration, measured by UV‐visible spectrophotometry, was standardized to 2.5 mg/mL with buffered saline before boiling in 1× Laemmli buffer with 2‐mercaptoethanol (Biorad, Hercules, California) and placing on ice for 5 min. After treatment with protease inhibitor (Halt, Thermo Fisher Scientific, Waltham, Massachusetts), samples were stored at −80°C and analyzed within 6 months of collection. The optimization and standardization of protein loading were established by sodium dodecyl sulfate‐polyacrylamide gel electrophoresis (SDS‐PAGE) and staining of SDS gel by Coomassie blue (Figure S1). A constant quantity of plasma proteins (43.75 μg or 1.25 mg/mL) from each subject then was separated by SDS‐PAGE before transfer to polyvinylidene fluoride membranes (Biorad, Hercules, California). Membranes were stained with 0.1% Ponceau S (Sigma‐Aldrich, St. Louis, Missouri) to confirm adequate transfer of proteins and then blocked in 10% bovine serum albumin (Fisher Scientific, Pittsburgh, Pennsylvania; overnight, 4°C) before incubation with a rabbit polyclonal anti‐human citH3 antibody (1:2000, ab5103, Abcam, Cambridge, Massachusetts; 2 hours, room temperature), previously shown to cross react in cats.^20^ After washing 5 times in 1× tris‐buffered saline with 2% Tween (TBST), membranes were incubated in goat anti‐rabbit secondary antibody conjugated to horseradish peroxidase (1:20000, Abcam, Cambridge, Massachusetts) for 1 hour at room temperature. Chemiluminescent substrate (WesternBright Quantum, Advansta, San Jose, California) was added directly on the blots and imaged (Fluorchem E, Protein Simple, San Jose, California). To confirm loading and cross‐reactivity of anti‐citH3 antibody in cats, a serial dilution of human recombinant citH3 (0, 1.95, 3.9, 7.8, 15.6, 31.25, 62.5, 125, and 250 ng/mL) was prepared in commercially available heparinized pooled feline plasma, DNA‐digested (BioChemed Services, Winchester, Virginia). Pooled heparinized feline plasma (BioChemed, Winchester, Virginia) also was subjected to sonication and DNA digestion as described above and denatured, reduced and underwent immunoprobing along with patient plasma samples. Densitometry was performed using available software (ImageJ, NIH), which then was used to generate a standard curve for each blot, extrapolated based on nominal log concentrations of human recombinant citH3. The suitable linear interval (densitometry: 4245 to 30 665) was used to interpolate the standard curve by utilizing the best‐fit linear equation, y=mx+b. Incubation times, and dilution of antibodies were optimized in preliminary experiments. A dilution factor of 3 was used to measure the final concentration of citH3 in all samples.

### 2.8 Statistical analysis

Sample size calculation was performed based on preliminary data and an anticipated difference of 25%, an 80% power, and a priori alpha of 0.05. Because cats in the CATE group were expected to have substantially higher cfDNA concentrations, an unequal sample size comparison was selected with at least 15 cats with an anticipated difference of 35% and 90% power when compared to non‐HCM or HCM groups. Normality was tested using the Shapiro‐Wilk normality test. Normally distributed continuous data, presented as mean ± SD, were analyzed using *t* test or 1‐way ANOVA, followed by post‐hoc analysis using Tukey's multiple comparisons test. Nonparametric data, presented as median and interquartile range (IQR), were analyzed using the Mann‐Whitney or Kruskal‐Wallis test followed by post‐hoc analysis by Dunn's multiple comparisons test. Categorical data between 2 and 3 groups were compared using Fisher's exact test and Chi‐squared test, respectively. Pearson correlation coefficients were calculated to describe the relationship between selected echocardiographic results, total cfDNA concentration and citH3 densiometry results, as well as the relationship between cfDNA, and citH3 and neutrophil count from the CBC. Statistical analysis was performed using commercially available software (Graphpad Prism 8). A *P* value <.05 was considered significant.

## 3 RESULTS

### 3.1 Animals

Eighty‐five cats were evaluated for enrollment at the Veterinary Medical Teaching Hospital, University of California, Davis from July 2019 to January 2021. Baseline demographics, hematological and biochemical findings are summarized in Table 2. Of the 17 cats with CATE, 7 were presented with rectal temperature < 37.6°C, and 12/17 (70.6%) cats had more than 1 limb affected. Six cats (42.9%) had documented hyperkalemia (>5.8 mmol/L) on presentation. Eleven cats (64.71%) were euthanized shortly after the diagnosis of CATE and study enrollment. Of the 8 cats that were hospitalized, 4 cats (50%) survived to discharge. The remaining 4 cats were euthanized because of the development of refractory hyperkalemia and acute kidney injury. Complete echocardiography was not performed in 2/17 cats with CATE because of patient instability. Table 3 summarizes the echocardiographic findings in the 3 groups. Of the 33 cats in the HCM group, 6 (18.18%) were in CHF at the time of enrollment and 3 (9.09%) had evidence of SEC or intracardiac thrombosis or both with the remainder of the cats in this group being subclinical for their HCM.

**TABLE 2:** Summary of baseline demographic information for 85 cats.

	Non‐HCM (N = 35)	HCM (N = 33)	CATE (N = 17)
Male	20 (57%)	21 (61%)	11 (61%)
Female	15 (43%)	11 (32%)	7 (37%)
Age (year)	6.31 ± 4.52	6.43 ± 3.63	7.36 ± 3.04
Weight (kg)	4.82 ± 0.92	5.12 ± 1.28	4.86 ± 1.31
Hematology			
RBC (×10^9^/L)	8.8 ± 1.3	9.0 ± 1.7	8.2 ± 1.4
White blood cells* (×10^9^/L)	7.2 (5.5 to 9.7)	9.3 (6.4 to 11.3)	11.0 (8.9 to 15.5)^a^
Neutrophils (×10^9^/L)*	4.1 (2.9 to 6.4)	6.8 (5.0 to 7.7)^b^	9.0 (6.5 to 11.8)^a^
Lymphocytes (×10^9^/L)	2.1 (1.3 to 3.6)	1.6 (1.2 to 7.3)	1.2 (0.9 to 2.4)
Platelets (×10^9^/L)	172.0 (IQR 121.8 to 216.3)	230.0 (IQR 118 to 279.0)	177 (IQR 112 to 239.0)
Mean platelet volume (fl)*	11.3 (IQR: 10.6 to 12.5)	10.5 (IQR: 9.9 to 12.0)	12.5 (IQR: 11.0 to 12.0)^c^
Biochemistry			
Potassium (mmol/L)*	4.1 (IQR: 4.0 to 4.5)	4.2 (IQR: 3.8 to 4.4)	4.6 (IQR: 4.3 to 6.6)^a^ ^,^ ^c^
Creatinine (mg/dL)	1.3 (IQR: 1.1 to 1.6)	1.4 (IQR: 1.1 to 1.8)	1.7 (IQR: 1.0 to 2.0)
BUN (mg/dL)*	24.0 (IQR: 22.0 to 28.5)	25.0 (IQR: 21.0 to 29.5)	40.0 (IQR: 23.0 to 49.0)^a^ ^,^ ^c^
Total protein (g/dL)	7.4 ± 0.6	7.3 ± 1.2	7.7 ± 1.0

^a^ CATE vs Non‐HCM.

^b^ HCM vs Non‐HCM.

^c^ CATE vs HCM.

* Significance between groups (*P* < .05).

**TABLE 3:** Summary of echocardiographic findings.

	Non‐HCM (n = 35)	HCM (n = 33)	CATE (n = 17)
2D IVSd (mm)*	4.4 ± 0.6	5.9 ± 1.2^a^	6.6 ± 2.2^b^
2D LVPWd (mm)*	4.8 (IQR: 4.4 to 5.3)	6.4 (IQR: 5.9 to 7.0)^a^	6.6 (IQR: 6.3 to 7.9)^b^
M mode IVS (mm)*	4.48 ± 0.56	5.79 ± 1.18^a^	5.62 ± 3.18^b^
M mode LVPw (mm)*	4.4 (IQR: 4.0 to 4.9)	6.0 (IQR: 5.3 to 6.9)	6.9 (IQR: 5.1 to 8.6)^b^ ^,^ ^c^
LA:Ao*	1.3 (IQR: 1.3 to 1.4)	1.4 (IQR: 1.3 to 2.2)	2.3 (IQR: 1.9 to 2.7)^b^ ^,^ ^c^
Left atrial enlargement*	‐	9/33 (27.27%)	17/17 (100%)
Left auricular velocity (cm/s)*	43.0 (IQR: 40.8 to 53.1)	53.6 (IQR: 41.7 to 71.3)	16.4 (IQR: 13.5 to 25.8)^b^ ^,^ ^c^
Fractional shortening (%)	58.2 (IQR: 47.0 to 63.4)	54.0 (IQR: 47.9 to 61.6)	47.4 (IQR: 40.8 to 53.7)
Spontaneous echocardiographic contrast*	‐	6/33 (18.2%)	8/15 (53.3%)
Intracardiac thrombus*	‐	3/33 (9.1%)	8/15 (53.3%)
Congestive heart failure*	‐	6/33 (18.2%)	12/15 (70.6%)

^a^ HCM vs Non‐HCM.

^b^ CATE vs Non‐HCM.

^c^ CATE vs HCM.

* Significance between groups (*P* < .05).

### 3.2 Cats with CATE have increased cell‐free DNA

**FIGURE 2:**
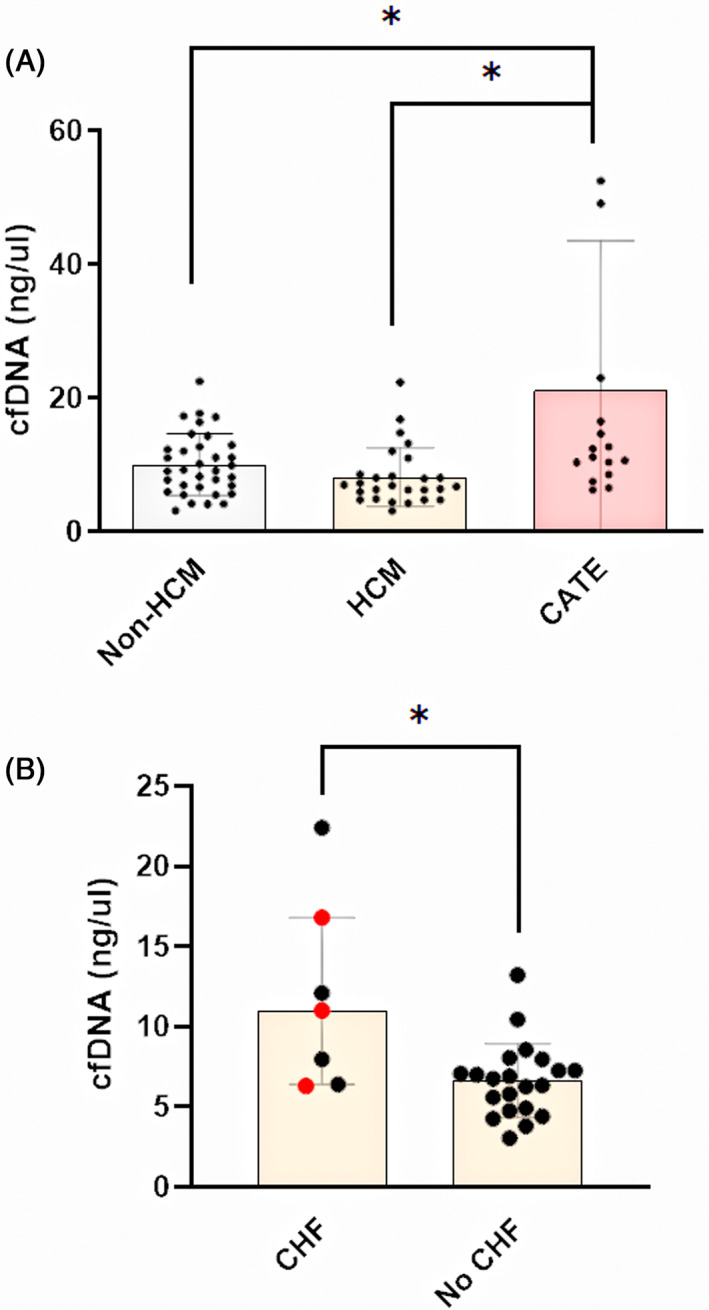
Plasma cell‐free DNA (cfDNA) concentrations measured by spectrophotometry in 34 cats of the non‐hypertrophic cardiomyopathy (HCM) group, 28 cats in the HCM group and 17 cats with HCM and cardiogenic arterial thromboembolism (CATE). (A) Cats with CATE had significantly higher concentrations of plasma cfDNA compared to cats with or without HCM. (B) Cats in congestive heart failure (CHF) secondary to HCM were found to have higher plasma cfDNA concentrations than those without CHF. Red dots indicate cats with intracardiac thrombus and/or spontaneous echo‐contrast. Bar represents median and error bars represent interquartile ranges. *P < .05.

Plasma from 79 cats was available for cfDNA analysis by spectrophotometry (CATE = 17, Non‐HCM = 28, HCM = 34). Samples from 4 cats in the non‐HCM group were not included because of technical errors that occurred during the isolation and purification process. One cat in the HCM group was excluded because of sample loss. Overall, cats with CATE had increased cfDNA (11.2 ng/μL; IQR, 8.1 to 29.6) compared to those in the HCM (6.6 ng/μL; IQR, 5.1 to 8.3; *P* = .0003) and non‐HCM groups (8.2 ng/μL; IQR, 5.7 to 11.7; *P* = .01; Figure 2A). Subgroup analysis showed that cats in the HCM group with concurrent CHF had higher plasma cfDNA concentrations compared to those without CHF (11.0 ng/μL; IQR, 6.4 to 16.8 vs 6.3 ng/μL; IQR, 4.8 to 7.3; *P* = .01; Figure 2B).

### 3.3 Cell‐free DNA size distribution profiles differ among cats with CATE

**FIGURE 3:**
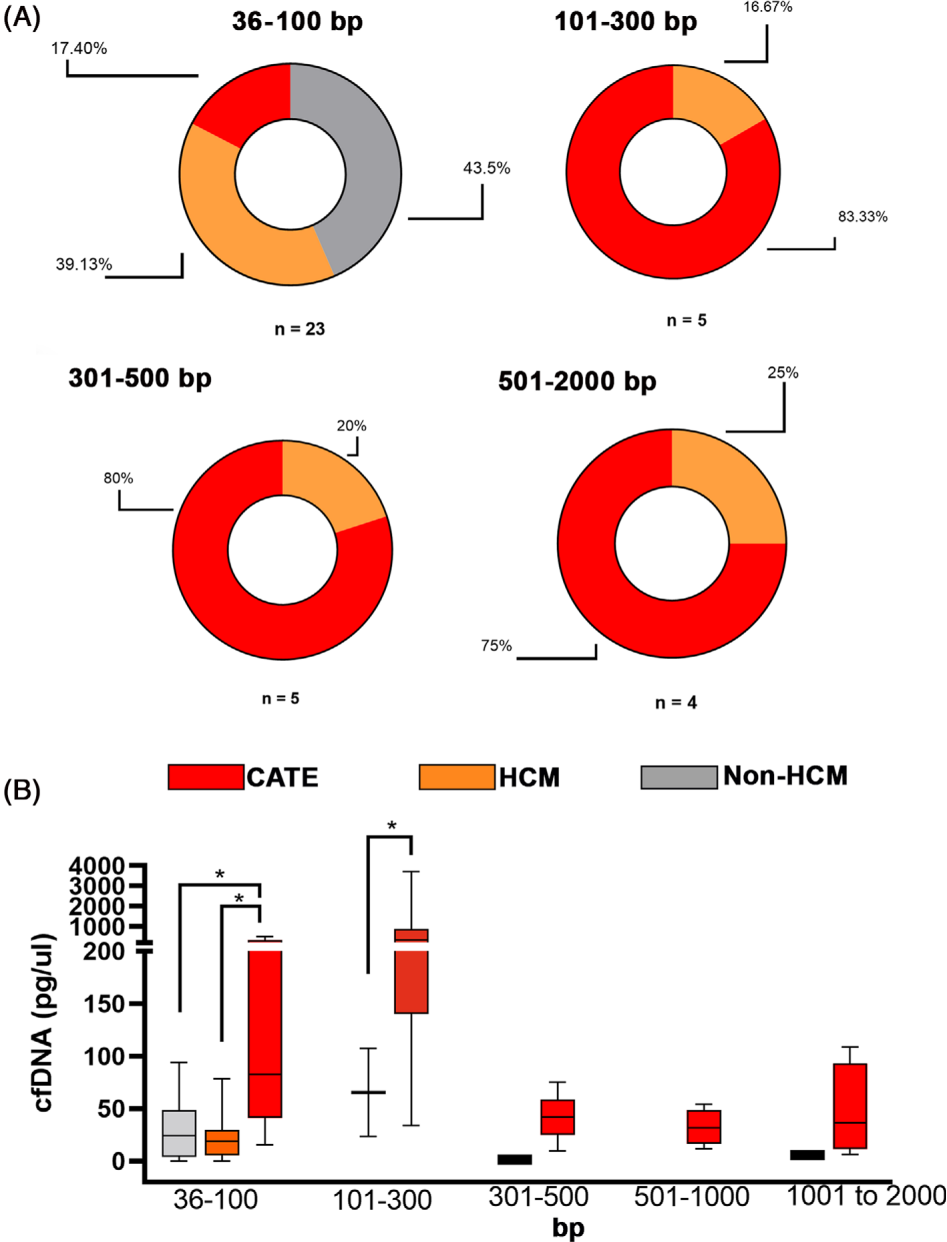
Proportion of cats grouped according to detectable cell‐free DNA (cfDNA) fragment sizes, 36‐100 base pairs (bp), 101‐300 bp, 301‐500 bp, or 501‐2000 bp using high‐sensitivity electrophoresis. (A) Only cats with CATE or hypertrophic cardiomyopathy (HCM) had detectable cfDNA fragments between 100 and 2000 bp. (B) Distribution of mean cfDNA concentrations grouped based on cfDNA fragment size and groups. Cats with CATE had significantly higher cfDNA at 36‐100 bp and 101‐300 bp compared to cats in the HCM group. *P < .05.

To further characterize cfDNA fragment sizes, chip‐based microfluidic electrophoresis was utilized. Representative electropherograms are shown in Figure 1. Of the 78/79 samples available for further analysis, cfDNA fragment size >2000 bp was detected in 35 cats (44.9%) whereas fragment size <2000 bp was identified in 71 cats (91.03%). Overall, the proportion of cats with cfDNA <2000 bp was significantly higher in cats with CATE (16/17, 89%) compared to those in the HCM (12/23, 52%; *P* = .02) and non‐HCM (12/31, 38.71%; *P* = .0008) groups. Figure 3A presents the proportion of cats grouped based on their cfDNA size profile. No cats in the non‐HCM group had detectable cfDNA fragments sized between 101 and 2000 bp. Cats in the CATE group were responsible for 75% to 83% of cases with detectable cfDNA fragments between 100 and 2000 bp whereas cats in the HCM group were responsible for the remaining 17%‐25%. When cfDNA concentrations in the 3 groups were compared by fragment size distribution, cats with CATE had the highest concentration of cfDNA between 36 and 100 bp (82 pg/μL; IQR: 41 to 3132; *P* = .02) compared to non‐HCM controls (19 pg/μL; IQR, 5 to 30; *P* = .02) and cats with HCM (24 pg/μL; IQR, 4 to 49; *P* = .03). Additionally, cats with CATE had a significantly higher concentration of cfDNA between 101 and 300 bp compared to cats with HCM (332 pg/μL vs 65 pg/μL; *P* < .001; Figure 3B).

### 3.4 Cats with HCM and CATE have increased concentrations of plasma citrullinated histone H3

**FIGURE 4:**
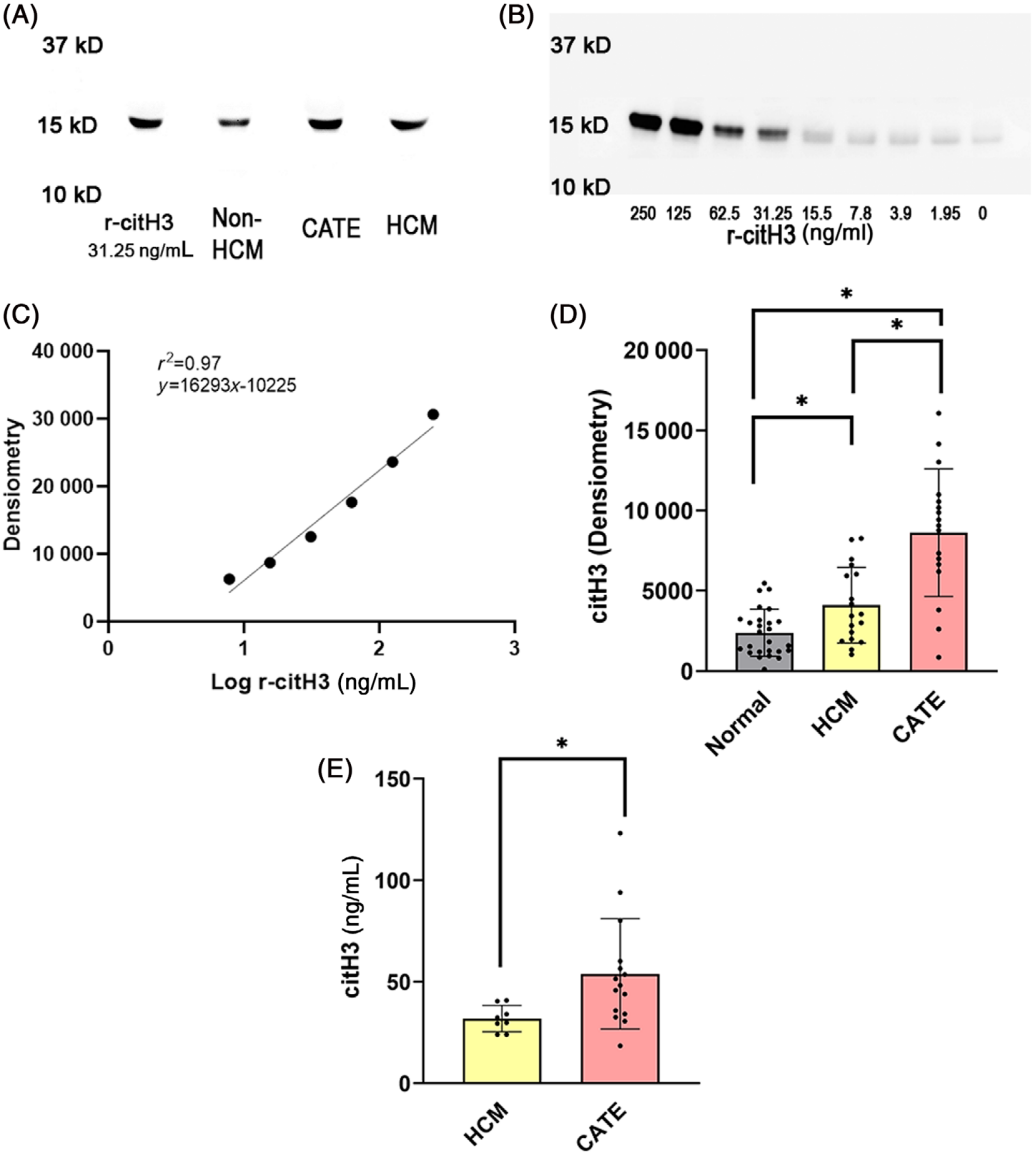
Plasma citrullinated histone H3 (citH3) analyzed by Western blot in 23 cats without (hypertrophic cardiomyopathy [HCM]), 21 cats with HCM and 16 cats with cardiogenic arterial thromboembolism (CATE). (A) A representative immunoblot of human recombinant citH3 (r‐citH3) (31.25 ng/mL), plasma from a cat without HCM, a cat with CATE, and a cat with HCM demonstrating identical molecular weight of 15 kDa and cross‐reactivity with anti‐human citH3 antibody. (B) A representative immunoblot of human recombinant citH3 (r‐citH3) proteins diluted to various concentrations in 50% pooled feline plasma. (C) Densitometry (arbitrary unit) was used to generate a representative standard curve for quantifying plasma citH3 concentration in feline patients. (D) Cats with HCM and CATE had significantly higher plasma levels of citH3 based on densitometry compared to cats without HCM. Those with CATE had higher citH3 than those with HCM. (E) After converting the densitometry signals to concentrations, none of the cats without HCM and 11 cats with HCM had plasma citH3 levels above the lower limit of detection (23.2 ng/mL). Plasma citH3 concentration in cats with CATE was significantly higher than cats with HCM. Bar presents mean and error bars represent standard deviations. *P < .05.

We demonstrated cross‐reactivity and specificity of the anti‐human citH3 antibody in cats by first comparing immunoblots of human recombinant citH3 and feline plasma samples. Both human recombinant citH3 and feline citH3 migrated to the same molecular weight marker of approximately 15 kDa (Figure 4A). Representative immunoblots of plasma citH3 in cats from the 3 groups are shown in Figure 4A. To semi‐quantitatively measure the concentrations of patient citH3, a standard curve for each immunoblot was generated by serial dilution of human recombinant citH3 prepared in pooled feline plasma as shown in Figure 4B,C. Inadequate amount of plasma in 12 HCM cats, 12 non‐HCM, and 1 cat CATE prevented analysis of plasma citH3 in these cats. Overall, 60 cats, including 23 non‐HCM cats, 21 HCM cats and 16 cats with CATE were further analyzed for plasma citH3 concentrations using Western blot analysis. Plasma citH3 concentrations were significantly different among the 3 groups (*P* < .0001). Cats with HCM had higher concentrations of plasma citH3 (4101 ± 2350 ng/ml) compared to cats in the non‐HCM group (2380 ± 1254 ng/ml; *P* = .005) whereas cats with CATE had the highest plasma citH3 concentration (8618 ± 3978 ng/ml) compared to cats with (*P* < .0001) or without HCM (*P* < .0001; Figure 4D). After factoring in the lower limit of detection (23.2 ng/mL) based on linear standard curves generated from recombinant human citH3 (Figure 4C), we found that 23/23 cats (100%) in the non‐HCM group, 11/21 cats (52%) in the HCM group and 3/16 (18.8%) cats in the CATE group had citH3 concentrations that were below the lower limit of detection. The number of cats with citH3 concentrations below the lower limit of detection was significantly different among the 3 groups (*P* < .0001). Plasma citH3 concentrations in cats with CATE remained significantly higher than those in cats with HCM (48.2 ng/mL; IQR, 34.2 to 60.2 vs 31.1 ng/mL; IQR, 25.3 to 38.8; *P* = .007; Figure 4E).

### 3.5 Plasma citrullinated histone H3 and cfDNA correlate with selected echocardiographic variables

**FIGURE 5:**
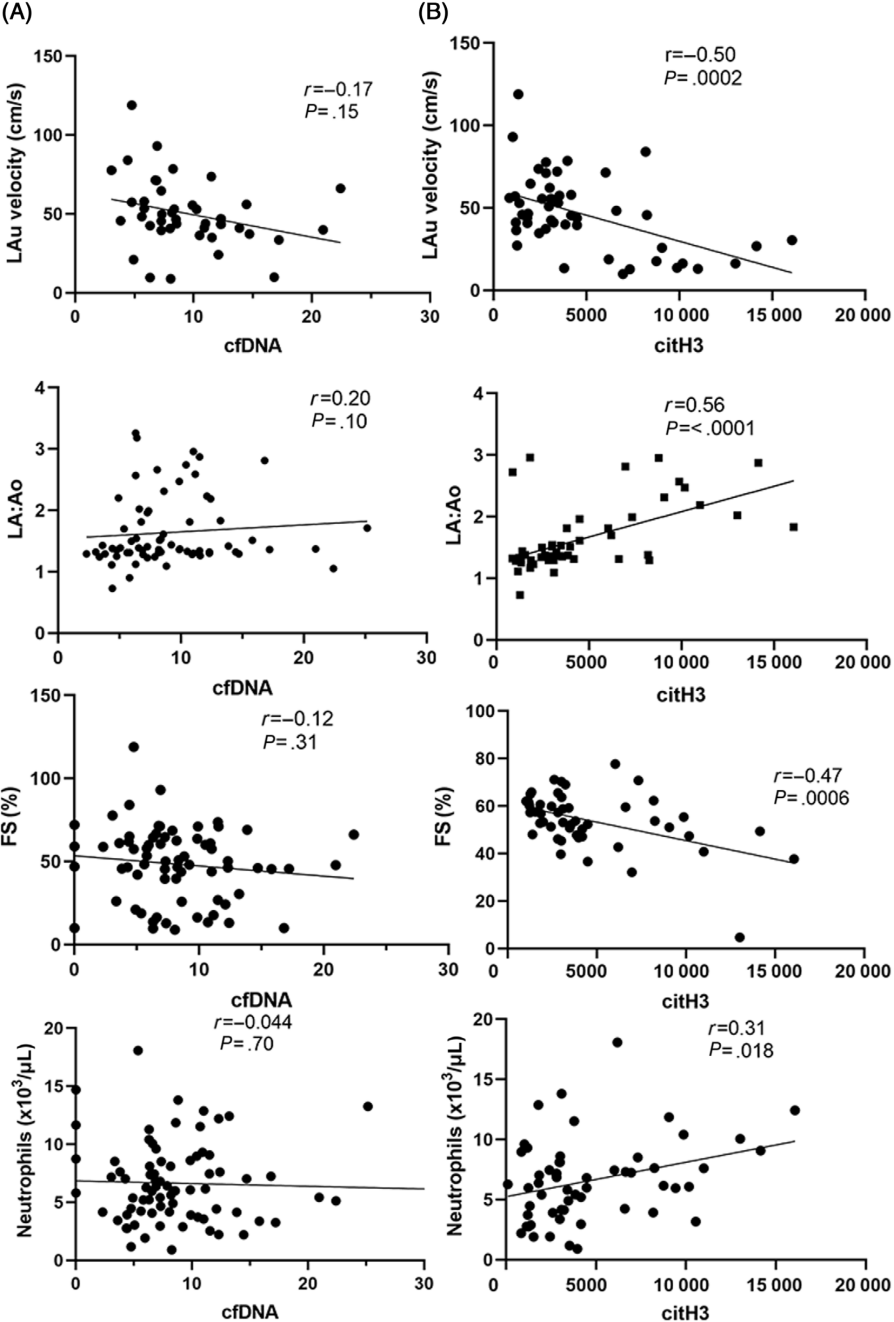
Plasma citrullinated histones H3 (citH3) correlate with predisposing factors to thrombosis in cats. (A) Cell‐free DNA (cfDNA, ng/μL) did not correlate with neutrophil count or other echocardiographic variables. (B) In contrast, negative and significant correlations were found among plasma citH3 (densiometry), LA function, measured as LAu velocity and left ventricular fractional shortening (FS%). Plasma citH3 was positively correlated with LA to aortic root ratio (LA:Ao) and neutrophil count.

No significant correlations were found between circulating cfDNA and left auricular flow (LAu) velocity (*r* = −0.17; 95% confidence interval [CI] −0.39 to 0.064; *P* = .15), LA:Ao (*r* = 0.20; 95% CI, −0.078 to 0.37; *P* = .10), LV fractional shortening (FS%; *r* = −0.12; 95% CI, −0.35 to 0.12; *P* = .31) or neutrophil count (*r* = −0.044; 95% CI, −0.26 to 0.18; *P* = .70; Figure 5A).

We found significant negative correlations between plasma citH3 and LAu velocity (*r* = −0.50; 95% CI, −0.69 to −0.26; *P* = .0002), and fractional shortening (*r* = −0.47; 95% CI, −0.66 to −0.22; *P* = .0006). Plasma citH3 was most strongly correlated with LA:Ao (*r* = 0.56; 95% CI, 0.34 to 0.72; *P* < .0001) and weakly correlated with neutrophil count (*r* = 0.31; 95% CI, 0.058 to 0.53; *P* = .018; Figure 5B). Interestingly, plasma cfDNA did not correlate significantly with citH3 (*r* = −0.09; *r* ^2^ = 0.008; *P* = .7). However, when cfDNA concentrations were grouped according to bp, a moderate and significant correlation was found between plasma citH3 concentration and cfDNA <2000 bp (*r* = 0.44; 95% CI, 0.17 to 0.66; *P* = .002). This determination was made only in patients with both measurements of citH3 and fragment size reported for cfDNA. Finally, citH3 concentration did not correlate with mean cfDNA fragments ≥2000 bp (*r* = −0.024; 95% CI, −0.31 to 0.27; *P* = .87).

## 4 DISCUSSION

We found that circulating NETs markers were significantly increased in cats with HCM and CATE. We also found that citH3 correlated significantly with predisposing risk factors of intracardiac thrombosis and thromboembolism in cats with HCM.

NETosis, a term that describes the active cellular processes underlying the formation of NETs, is dependent on complex and coordinated signaling facilitated by extracellular stimuli such as pathogens, danger‐associated molecular patterns, cytokines, reactive oxygen species and, most importantly, platelet‐neutrophil interactions.^30^, ^31^, ^32^, ^33^ Although the underlying mechanisms of NETosis in cardiomyopathies remain poorly understood, evidence in animal models of carotid artery thrombosis suggest that NETs formation is dependent on platelet activation, histone citrullination and upregulation of the neutrophil integrin, α9β1.^34^, ^35^, ^36^ These findings suggest that neutrophils in cats with HCM and CATE may be activated at the site of endothelial injury or via platelet‐neutrophil interactions resulting in NETosis. Given that NETs previously were found to be a component of arterial thrombi in cats, the presence of circulating NET markers in plasma further supports the notion that the bidirectional feedback of inflammation and thrombosis may contribute to a prothrombotic state that further promotes thrombosis.^21^

We found that circulating cfDNA was significantly higher in cats with CATE but did not correlate with selected risk factors of thrombosis on echocardiogram. There are several explanations for these findings. First, quantification of total cfDNA could not discriminate between genomic DNA contamination originating from necrotic muscle cells associated with CATE and secondary ischemic injury or active release of cfDNA from living cells. In addition, in vitro processing also could lead to cell lysis and subsequent genomic DNA contamination. Although every effort was made to minimize genomic DNA contamination by standardizing pre‐analytical variables such as venipuncture sites, blood collection techniques, sample processing and duration of storage, DNA fragmentation or degradation also may negatively affect the quality of the samples. Second, the variable volume of plasma from some cats may decrease the quality or amount of cfDNA isolated. To further determine the origin of cfDNA, DNA size profiling by high‐sensitivity electrophoresis was performed, which indicated that 50 to 60% of cats with or without HCM did not have detectable cfDNA fragments <2000 bp. Based on this observation, high‐sensitivity electrophoresis alone appears to be insufficient to characterize the size profile of cfDNA in most HCM and healthy cats. This finding suggests that high‐sensitivity electrophoresis should be coupled with amplification methods such as massive parallel sequencing and quantitative real‐time PCR.^37^ Still, the size profile of 100 to 500 bp, with a maximum peak at 167 bp, found in cats with CATE and HCM indicates that cfDNA in these cats likely originated from living cells because this pattern closely mirrors that found in nucleosomes.^38^ This finding also indicates that the increased cfDNA found in cats with CATE is secondary to an active process, most likely from NETosis, as opposed to necrotic tissues, which release long fragments of cfDNA >10 000 bp. Such findings have important clinical implications because cfDNA can serve as a novel therapeutic target for the prevention and treatment of cardiogenic thrombosis. For instance, cfDNA not only strengthens clot density by binding to erythrocytes and platelets, its poly‐anionic surface also binds to von Willebrand factor (vWF) molecules. Together, this DNA‐vWF interaction may play a crucial role in entrapping leukocytes and further propagating clot formation at the site of vascular injury.^39^ In addition, ischemic stroke models in mice found that lysing of cfDNA with DNase I not only facilitated recanalization of occluded arteries but also decreased tissue plasminogen activator‐associated hemorrhage.^40^, ^41^ Considering that thrombolytic treatment largely has failed to improve outcomes in cats with CATE, more research is needed in targeting cfDNA.^42^

In contrast to plasma cfDNA, citH3 was significantly increased in cats in the HCM as well as the HCM and CATE groups. In addition, all non‐HCM cats had citH3 concentrations below the limit of detection whereas approximately 50% of cats with subclinical HCM had increased plasma citH3. This finding is not surprising considering that citH3 is a more specific NETs marker.^43^ Citrullination of histones, which is a form of histone modification, is facilitated by the enzyme peptidyl arginine deiminase 4 (PAD4) across multiple species, but has not been characterized in cats^44^ The increase in plasma citH3 in our study suggests that PAD4, which is highly expressed in neutrophils, may be activated in cats with cardiomyopathy. In other species, PAD4 converts arginine residues to citrulline, thereby altering the electrostatic interactions between histones and DNA, resulting in chromatin decondensation and the subsequent release of DNA and NETs.^45^ In dogs, NETs formation induced by reactive oxygen species‐dependent pathways via phorbal myristate acetate or lipopolysaccharide is dependent on PAD4.^28^ Although increased PAD4 activity in neutrophils is essential for NETosis in other species, the underlying mechanisms mediating NETs formation and histone citrullination in cats remain unclear. Additional studies are needed to investigate if histones or upregulation of PAD4 could serve as potential therapeutic targets for preventing CATE in cats with HCM.

Plasma citH3 also correlated moderately and significantly with probable predisposing risk factors of cardiogenic thrombosis in cats with HCM. This observation suggests that plasma citH3 may be a biomarker in predicting thrombosis and CATE in cats. Considering that no healthy cat had increased citH3 concentrations, we believe increased citH3 is a marker of clinical concern. Currently, veterinarians have limited tools to recognize cats at risk of CATE because existing methods of identifying risk factors rely solely on echocardiographic findings such as presence of SEC, LA enlargement and LAA dysfunction. This identification is complicated by the fact that many cats at risk for CATE are apparently healthy and do not have auscultatory abnormalities, hence they are unlikely to be selected for echocardiographic screening.^5^, ^8^ In human beings, a number of studies indicate that increased citH3 is associated with thrombotic risk in cardiovascular disorders such as ischemic stroke, atrial fibrillation and myocardial infarction.^46^, ^47^, ^48^ Thus, measurement of free circulating citH3 maybe an accessible way to determine cats at risk of CATE. Unfortunately, our small sample size and an inadequate amount of residual plasma for citH3 measurements in some cats prevented us from performing subgroup analyses to further explore the associations between increasedcitH3 and prothrombotic risk factors. Additonal studies are needed to assess the diagnostic utility of plasma citH3 in cats with HCM so that thromboprophylaxis can be administered sufficiently early to prevent CATE. Although histone H3 is highly conserved among species and the feline protein is found to be 100% homologous to the human amino acid sequence, we noticed that feline plasma proteins consistently interfere with the detection of human protein standards in ELISA kits. This interference may be caused by nonspecific binding to the detection or capture antibodies or formation of histone‐plasma protein complexes. Given the limitations of Western blot analysis such as long processing times and its semi‐quantitative nature, custom development of a feline‐specific citH3 ELISA is a possible future method to accurately measure citH3 concentrations as a clinical test.

Our study had several limitations. First, although chip‐based capillary electrophoresis provides a reasonable estimation of cfDNA concentration, the small amount of cfDNA might have represented false negatives and this data should be interpreted with caution. More precise cfDNA fragment concentrations will need to be obtained by digital droplet PCR in future studies.^49^ Second, although correlations among NETs, especially in free citH3, were found in cats with HCM and CATE, NETs could be a marker of disease severity. The latter would require a longitudinal study in HCM cats characterizing both citH3 and cfDNA fragment size using high‐sensitivity detection and represents a future direction for this work. Third, we did not assess coagulation status of our study population and hence could not confirm the associations between circulating NETs and thrombosis in cats. Finally, inadequate volume of plasma samples and the semiquantitative nature of citH3 measurements prevented advanced subgroup analyses to further assess the diagnostic and prognostic utility of circulating citH3. Hence development of a feline‐specific assay to measure plasma citH3 reliably and accurately in cats is critically needed.

## 5 CONCLUSION

We found that cats with HCM and CATE had increased concentrations of circulating NETs, in the form of cfDNA at 100 to 300 bp and citH3. Approximately 40% and 80% of cats with HCM and CATE, respectively, had detectable plasma citH3, which correlated with thrombotic risk factors.

## CONFLICT OF INTEREST DECLARATION

Johaua A Stern serves as Associate Editor for the Journal of Veterinary Internal Medicine. He was not involved in review of this manuscript. No other authors declare a conflict of interest.

## OFF‐LABEL ANTIMICROBIAL DECLARATION

Authors declare no off‐label use of antimicrobials.

## INSTITUTIONAL ANIMAL CARE AND USE COMMITTEE (IACUC) OR OTHER APPROVAL DECLARATION

Approved by the University of California, Davis, IACUC, approval number 21303.

## HUMAN ETHICS APPROVAL DECLARATION

Authors declare human ethics approval was not needed for this study.

## Supporting information


**Figure S1.** Coomassie Blue‐stained SDS gel with standardized plasma protein concentrations (mg/mL) from a cat with cardiogenic arterial thromboembolism (CATE) and a healthy control cat to ensure consistency with loading for Western blot analysis. A plasma protein of 43.75 μg (1.25 mg/mL) was chosen for the optimal detection of free histone proteins (arrows).
