# Supplementary figures and images for: Circulating neutrophil extracellular traps in cats with hypertrophic cardiomyopathy and cardiogenic arterial thromboembolism
Source: J Vet Intern Med. 2023 Mar 23;37(2):490–502. doi: 10.1111/jvim.16676 (PMC10061180; doi:10.1111/jvim.16676)

CATE

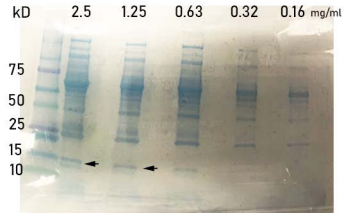

Normal

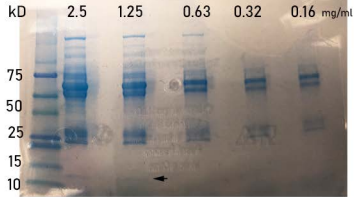

Supplement: Supplementary file 1 — Figure S1. Coomassie Blue‐stained SDS gel with standardized plasma protein concentrations (mg/mL) from a cat with cardiogenic arterial thromboembolism (CATE) and a healthy control cat to ensure consistency with loading for Western blot analysis. A plasma protein of 43.75 μg (1.25 mg/mL) was chosen for the optimal detection of free histone proteins (arrows). [file JVIM-37-490-s001.pdf]
